# Canine-Assisted Interventions and the Relevance of Welfare Assessments for Human Health, and Transmission of Zoonosis: A Literature Review

**DOI:** 10.3389/fvets.2022.899889

**Published:** 2022-06-17

**Authors:** Lieve Lucia Meers, Laura Contalbrigo, William Ellery Samuels, Carolina Duarte-Gan, Daniel Berckmans, Stephan Jens Laufer, Vicky Antoinette Stevens, Elizabeth Ann Walsh, Simona Normando

**Affiliations:** ^1^BIAAT Foundation, Genk, Belgium; ^2^National Reference Centre for Animal Assisted Interventions, Instituto Zooprofilattico, Legnaro, Italy; ^3^Hunter College, School of Nursing, The City University of New York, New York, NY, United States; ^4^Department of Psychology, Faculty of Humanities and Educational Sciences, University of Jaén, Jaén, Spain; ^5^Department of Biosystems, Katholieke Universiteit Leuven, Dier en Mens, Leuven, Belgium; ^6^Cork Pet Behaviour Centre, Cork, Ireland; ^7^Department of Comparative Biomedicine and Food Science, University of Padua, Padua, Italy

**Keywords:** canine-assisted interventions, welfare, zoonosis, preventative medicine, human animal interaction (HAI)

## Abstract

CAIs (canine-assisted interventions) include “canine-assisted therapy” in which a therapist sets client-oriented goals, 'canine-assisted activities' with recreational goals for clients, and 'canine-assisted education/learning' in which teachers or coaches create learning goals for students or clients. CAIs vary in nearly every way; their only common trait is the involvement of dogs to respond to human need. However, the benefits of involving dogs are highly dependent on the animal's health and behavior. A dog exhibiting negative behavior or an unwell dog might pose a risk, especially for CAI target groups, specifically individuals with immunosuppression, chronic illness, children, elderly, etc. Therefore, positive animal welfare as preventative medicine to avoid incidents or transmission of zoonosis is an attractive hypothesis, with implications for human and animal, health and well-being. This review aims to summarize the current published knowledge regarding different aspects of welfare in CAIs and to discuss their relevance in the light of health and safety in CAI participants. As method for this study, a literature search was conducted (2001–2022) using the Prisma method, describing issues of dog welfare as defined in the Welfare Quality^®^ approach. This welfare assessment tool includes 4 categories related to behavior, health, management, and environment; it was, therefore, applicable to CAIs. Results indicate that dogs working in CAIs are required to cope with diverse variables that can jeopardize their welfare. In conclusion, we propose regular welfare assessments for dogs in CAIs, which would also protect the quality of the CAI sessions and the clients' safety and well-being.

## Introduction

An increasing number of papers suggest that canine-assisted interventions (CAI) may have physical and psychological benefits for numerous target groups of varying ages and diagnoses (1–5). Neutral or positive impacts of CAI sessions on dog welfare are documented, based on physiological parameters (e.g., oxytocin levels, a negative feedback regulator that culminates with a decrease in cortisol, heart rate or blood pressure (6–17) and behaviors such as playing, leaning toward, nudging, sniffing or licking the client (18–22). The dog's temperament and individuality is seldom investigated, although it is likely that this influences physiological and behavioral outcomes (23).

Several studies report that the benefits of CAIs are greater than the risks (24–28), nevertheless some find potential stress or welfare risks in dogs a valid reason not to use CAIs (24, 29–34). Contra-indications for CAIs, include fear/phobia of animals, cultural attitudes (29), unsafe animal behavior (30, 35), allergic reactions (36), workload (37, 38), funding (39), concerns regarding hygiene/sanitization (39), or zoonotic transmission of diseases (40, 41).

Legislative frameworks such as the National Guidelines for AAI (42) in Italy stipulate that before joining CAIs, dogs must pass a veterinary assessment of their physical health, behavior, and welfare (43). The Austrian Ministry of Labor, Social Affairs, and Consumer Protection Initiative legally regulates therapy dogs, stipulating regular health/temperament/behavior checkups to re-evaluate an animal's suitability (44). In Germany, the integration of animals in AAIs is legally regulated, and animal handlers must provide evidence of their species-specific knowledge (45). In the USA, a few states have enacted public access laws for handlers with therapy animals comparable to that granted to service animal handlers (46). In other countries, the national and/or municipal animal protection/welfare legislation applies. Currently, no universally agreed welfare protocol exists (47, 48). Organizations that deliver CAIs and those that evaluate and register CAI dogs often have their own procedures for screening and instructing dogs and their handlers (49), posing a challenge to safe CAI sessions (50, 51).

This review aims to provide information on those welfare issues in CAI dogs that might cause health problems in CAI participants.

## Methods

**Figure 1:**
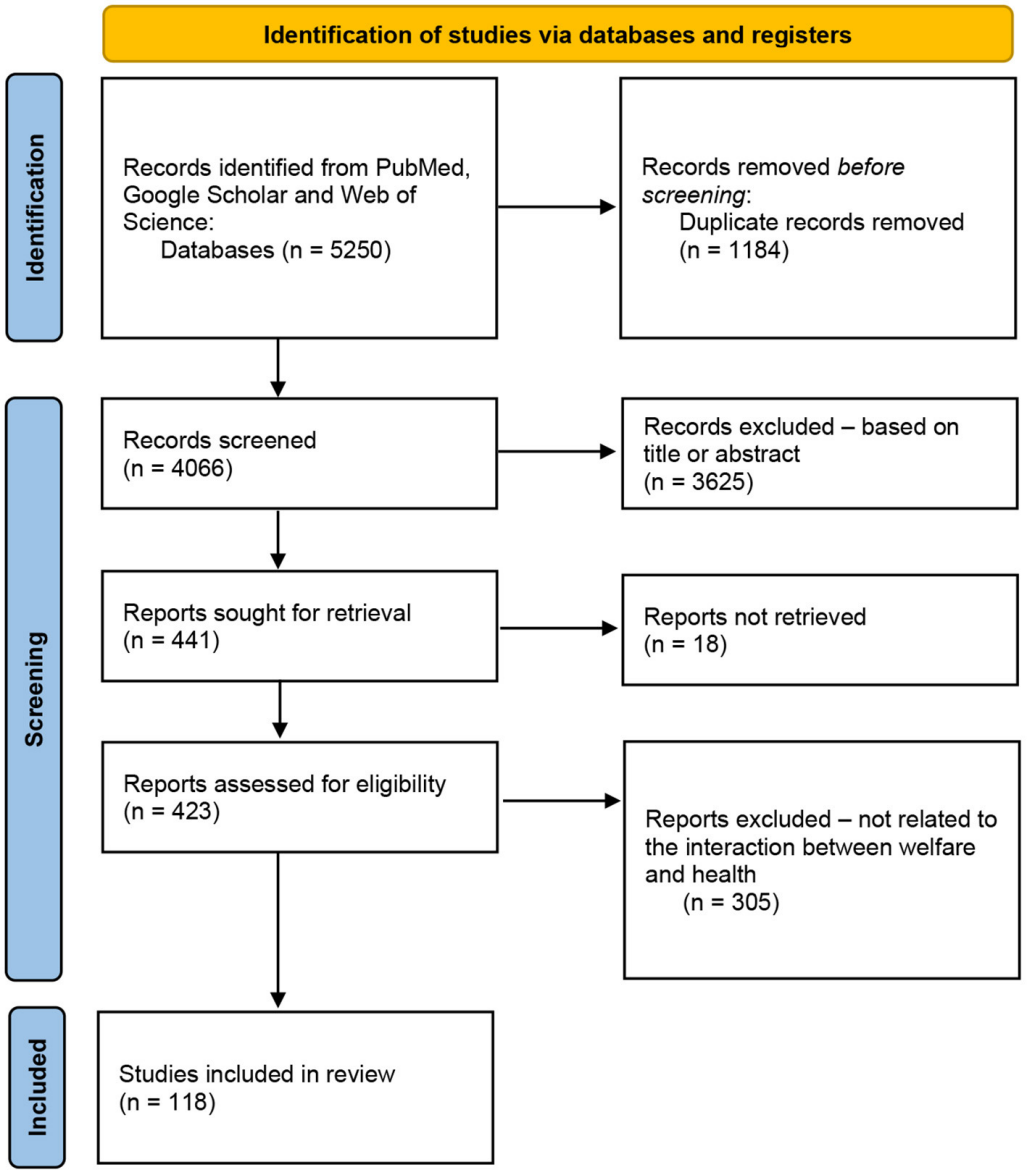
Flow diagram of the steps followed in the search strategy (53).

This review was conducted according to the protocol of the PRISMA group (52, 53). Only studies published between 2001–2022, a period during which CAIs increased prolifically, were selected to ensure a complete overview, while publications not written in English, case reports, (doctoral) theses, book chapters, conferences, commentaries, and notes were excluded. Publications reporting on welfare risks to dogs that might be related to physical problems in CAI participants were included; studies concerning assistance dogs were excluded. Three electronic scientific databases were searched: PubMed (54), Google Scholar (55), and Web of Science (56). The systematic search was performed in May 2022, by two of the authors (LM, CD-G) independently, using 5 strings (Table 1) in the Harzing's Publish or Perish software (57). The cut-off was set on 500/string (PubMed and Google Scholar) and 50/string (Web of Science). All terms were selected based on international reference guidelines for AAIs (58, 59) and the Welfare Quality model (60, 61). References from selected papers were revised and used as supplementary information sources. All data were entered into an Excel data set. We included data related to the welfare risks of dogs as described in the Welfare Quality model, possible physical consequences for CAI participants, and additional data to facilitate the identification of each study (e.g., authors, title, and year of publication). After excluding duplicates; titles and abstracts were evaluated (Figure 1). A total of 423 papers met the inclusion criteria and underwent full-text screening *via* Sci-Hub, OpenAccess, and DeepDyve. Finally, 118 articles were selected in consensus for relevance to the topic.

**Table 1:** Search string used in Google Scholar, Web of Science, PubMed.

(1) “animal-assisted therapy” OR “therapy dog” AND welfare (2) “animal-assisted therapy” OR “therapy dog” AND “raw meat” (3) “animal-assisted therapy” OR “therapy dog” AND zoonosis (4) “animal-assisted therapy” OR “therapy dog” AND “One Health” (5) “animal-assisted therapy” OR “therapy dog” AND abuse OR “dog bite”

## Welfare Quality^®^ Concept

### Management

Good management implies for example that dogs must have unlimited access to clean water, especially during CAI visits, as temperatures in nursing homes/clinics may be high (62). Also the appropriate quantity of food is important to avoid overfeeding, obesity and, if under-exercised, welfare issues such as congestive heart failure (63, 64). Obesity in CAI dogs is often related to food-rewarded activities/training.

Feeding a raw meat diet (RMD) is another contentious issue (50, 65, 66). RMD's include various dietary formats ranging from incomplete, unprocessed (i.e., unsterilized) foods to complete, balanced, sterilized diets (67). Proponents of RMD reference that these diets release maximum thermodynamic free energy (68) and increase the relative abundances of bacteria associated with protein and fat utilization, including members of the genera Fusobacterium and Clostridium. In humans, these genera are more commonly associated with disease (69). Some AAI institutional guidelines prohibit feeding RMDs, raw eggs, or raw treats such as dried pig ears to dogs participating in CAIs (49, 70) because these diets are linked to nutritional imbalance (70, 71), contagious viruses such as Pseudorabies (Aujeszky's disease) or (72), bacterial pathogens such as *Escherichia coli, Listeria* spp., *Clostridium, Salmonella* spp., *Campylobacter*, and parasitic pathogens such as *Cryptosporidium, Sarcocystis cruzi, Sarcocystis tenella, Toxoplasma gondii*, and *Neospora* (73–78). Additionally, a study by Finley et al. (79) showed that 7 out of 16 dogs fed RMDs contaminated with *Salmonella*, shed *Salmonella* serovars in their stools for up to seven days after consumption, even though the dogs did not subsequently exhibit clinical signs of disease. Recent reports linked RMDs to *Mycobacterium* (*M*.) *bovis*, which is one of the member species of the Mycobacterium tuberculosis complex (MTBC). It is not proven that dogs having had contact with mycobacterium *via* RMDs may transmit this zoonosis to humans, however, *M*. *bovis* is capable of causing tuberculosis across a broad taxonomy of species, including humans (80, 81).

Another welfare parameter that may be relevant is the food and drink that clients share with dogs during CAI activities. Clients may bring left-overs from their own meals to reward the dogs. However, some human foods contain components that are unhealthy or even toxic to dogs such as grapes, chocolate, or nuts (50, 62, 82). Moreover, feeding dogs means direct contact with dog saliva, a possible source of contact with commensal zoonotic pathogens resident in the dogs' oral cavities (62, 83). Such pathogenic species include *Pasteurella* spp. and *C*. *canimorsus*, which can cause infections, pneumonia, meningitis, or ulcerations in humans if licked in the ears, mouth, or in surgical wounds (84). Some institutions may therefore request that treats are deposited in a bowl to avoid direct contact between the client's hand and the dog's mouth (82). Regular disinfection of food and water bowls between CAI settings may be in place to reduce the risk of exchange of pathogens and to improve dog welfare during CAI sessions (82).

### Environment

CAIs are conducted in indoor facilities such as clinics, healthcare facilities, community centers, prisons, libraries, schools, retirement communities, homes, and outdoors in fields and forest (85). Welfare factors are influenced by the novelty of the environment, appropriate arrangements for canine specific morphology (47), access to a private/rest space, possibility to retreat out of view of clients, the frequency and duration of time spent in contact with clients (31).

As each environment has its own benefits and challenges, safety guidelines are seldom agreed upon. For example, in hospitals, wheelchairs and orthopedic equipment can pose a danger to animals (35); dogs might become entangled in IV bags and lines, (50) or accidently swallow medication (86). In outdoor settings, due to climate change, it may be important to note that ticks, are questing around in fields at air and soil temperatures as low as 2.5°C (87, 88). Tick bites are associated with the risk of catching Lyme disease sometimes with opportunistic co-infections such as chlamydia pneumonia, or mycoplasma transmissions (89). In recent years, there has been an increase in the number of case reports describing clinical tick-born encephalitis (TBE), a tick-borne viral disease, in dogs, with some coming from previously non-endemic areas, raising concerns. Currently, TBE vaccination is available for humans. First results indicate that human vaccines can be used in dogs but further studies are urgently needed (90).

Another challenge to meet for CAIs that are organized in woody areas is (in)direct contact (*via* dog saliva) with the hair of the oak and pine processionary caterpillar, a mechanic and toxic mechanism (lepidopterism) which can cause dermatitis, cutaneous reactions (weal and flare reaction), ocular lesions or upper respiratory tract reactions in humans. In dogs, this can cause labial angiooedema, ptyalism, sloughing, tongue swelling, stomatitis, conjunctivitis or respiratory distress (91–93).

Other ectoparasites such as mites and fleas (50) can cause health and welfare problems in dogs and humans if left untreated, and cause zoonosis such as *Bartonella* spp. (94) or flea allergy dermatitis in humans and dogs (95). Testing for endoparasites in dogs is also essential from a welfare perspective. Eggs, larvae, cysts, and oocysts excreted *via* the canine fecal route can survive and be infectious in the environment over a long period of time and under different conditions (96, 97). Dog feces deposited on soil in city parks/gardens are an inconvenience, and can pose a health threat such as the spread of Giardia or Toxocara eggs which can be transferred from animals to humans by fur contact (98–100). The effect of parasitic worms on clients can be complex, as they may have beneficial and/or adverse effects on clients' immune systems; an example being, definitive host helminth infections may confer protection from allergies however zoonotic helminths, such as *Toxocara* (spp.), may increase human allergy and asthma risks (101). *Toxocara* (spp.) can reproduce in dogs but not in humans; however, the larva can remain encapsulated in eggs for years in the human body. Yogi et al. (101) shows that young people who test positive for this parasite have a four times higher risk of developing asthma and allergies than others. Finally, some studies associate neurotropic parasitic diseases such as toxoplasmosis with mental disorders, and toxocariasis is associated with an increased risk of schizophrenia and/or bipolar disorders. People diagnosed with Chagas disease and/or neurocysticercosis have a higher risk of developing anxiety and depressive disorders (102). Therefore, CAI organizations ask that dogs involved in CAIs be tested annually by a veterinarian (negative stool and negative heartworm) and receive regular preventative treatment for parasites. Nevertheless, Gerardi et al. (103) showed a presence of four zoonotic parasites (nematodes and protozoa) in CAI dogs that were properly treated, demonstrating an urgent need for extra prevention measures.

Most protocols agree that dogs should be washed, and groomed within 24 h prior to contact with clients (49, 65, 66, 82). Washing a dog at least twice a week might be stressful for the animals (104), but it may help reduce Can f1 from dog hair and dander and lower the risk for allergic reaction (105). Allergies can cause skin irritations, allergic rhino-conjunctivitis, and allergic bronchial asthma. They are a contraindication for CAIs as they occur when in (in)direct contact with dogs (36).

Involving inappropriately termed hypoallergenic animals, such as labradoodles, does not provide an allergen-free environment (36). Moreover, Vredegoor et al. (106) found that Can f 1 in the hair and coat, a component that counts for at least half of the allergenic activity, was higher in these dogs than in control breeds such as Labrador retrievers. From a welfare aspect, the allergological risks of implementing CAIs in a hospital or outpatient setting should not be underestimated (36). Toshihiro et al. (107), shows that only the complete avoidance of animals was effective in patients with animal allergic asthma. Therefore, Schmidt et al. (36) states that emergency medication must be available to a trained person on site, to mitigate risk, in the event of an allergic response.

### Good Health

Some studies reveal that staff members oppose CAIs because of their fear of zoonosis (108–110). Lefebvre et al. (111) shows that transmission of zoonotic bacteria, viruses, or fungi between dogs and humans *via* infected saliva, aerosols, contaminated urine or feces, and direct contact is possible during CAIs. Therefore, most protocols request that dogs not exhibit indicators of poor health (e.g., vomiting, diarrhea, lethargy, etc.), take immunosuppressive medications or antibiotics during CAIs (49). Prior to starting CAI's, dogs should be in a permanent home for 6 months and be a minimum of 1 year/old. Puppies <16 weeks may be more susceptible to becoming sick as their immune systems are less strong than adult dogs (49) and more often carry parasites (112). However, some CAIs do involve puppies as they elicit a strong nurturing response from clients, and the handling experience of the visit may enhance the puppies' maturation and socialization (62). Many protocols request up-to-date vaccinations (rabies, parvovirus, distemper/canine adenovirus, leptospirosis, Bordetella, and canine influenza), and a yearly medical checkup by a veterinarian (49).

However, some studies (25, 97, 111) show that dogs judged to be in good health can become asymptomatic carriers of infection (e.g., Clostridium difficile, MDR bacteria). Other studies show dogs can carry methicillin-resistant Staphylococcus aureus (MRSA) (111, 113, 114) after visiting human healthcare settings. Only, Lefebvre et al. (111) claim that CAIs in hospitals did not result in increased nosocomial infection rates of zoonotic infections.

When addressing zoonotic diseases, prevention protocols focus mainly on the dogs. The most common viral and bacterial zoonotic infections transmitted to humans by dogs are viral infections such as rabies and norovirus, and bacterial infections including *Pasteurella, Salmonella, Brucella, Yersinia enterocolitica, Campylobacter, Capnocytophaga, Bordetella bronchiseptica, Coxiella burnetii, Leptospira, Staphylococcus intermedius, Streptococcus equi*, and *Methicillin-resistance Staphylococcus aureus* (114–118).

Animals always combine in a team with an animal handler, who may also carry zoonotic agents. In many guidelines, animal handlers are therefore presumed to follow the same rule as their animal; that is, not to visit their clients when displaying any symptoms of illness such as; fever, cough, diarrhea. Currently, humans are not required to have a yearly health screening from a physician or up-to-date vaccinations (49). The most reliable recommendation to safeguarding welfare and preventing the transmission of zoonotic disease is consistent hand hygiene and/or disinfection (25, 66, 82, 119) before and after visits and barrier protection such as linen disposal or changing the sheets on the bed after a visit (119). As animals often visit more than one setting, infection control tracking reports are also advisable (108).

### Appropriate Behavior

CAIs would not exist without dogs. It is crucial to ensure clients' well-being and animal welfare (120). Canine body language (47) (e.g., gaze, yawning, lip-smacking etc.,) (29, 121) is widely used to assess animal welfare (122) however, there is a paucity of investigation regarding the impact of handling on the welfare of CAI dogs. Some physical interactions that humans enjoy during CAIs, are not always perceived as pleasant by dogs, such as physical intimacy with strangers, being restrained on the lap, teased with food or toys, kissed on the muzzle/face (123), or stared at in the eyes (85). Moreover, a small percentage of animals sustain injuries during CAIs (124, 125) due to clients' rough handling. These human behaviors may cause stress in the dogs, undesirable snapping (126), or even bites (127, 128). Bite wounds have a special position in traumatology due to the risk of complications. Antibiotic therapy for infected wounds, tetanus immunization status, and rabies infection risk are needed in bite wound management (129). Tetanus in dogs is thought to be uncommon (130). However, Burkitt et al. (131) show that when dogs develop severe tetanus, and younger dogs were significantly more at risk, the clinical course of the disease is similar to that of severely affected humans. A similar relationship has been identified in humans, in whom tetanus results in death most frequently among the young and the elderly (130, 132).

Research on proximal causality and (legal) consequences of dog bites in AAIs is needed as the possibility of a dog bite may increase if a dog is in/on the clients' bed and may be motivated through fear and/or anxiety (127, 133). Good training and welfare protocols as well as the presence of a handler mitigating for every scenario (134) will assist in avoiding bite incidents. The current guidelines of a minimum age requirement for CAI recognize that puppies may exhibit less predictable behavior, which poses an increased risk for bites, falls, or scratches. Some protocols advise clipping the dog's nails before every visit to avoid scratches (130, 135). Additionally, it may be required to undertake behavior and temperament assessments using tests designed to simulate the circumstances of working environments prior to starting CAIs. Other guidelines request behavioral re-evaluation, every 2–3 years and/or the exclusion of certain breeds (e.g., trained guard-dogs, American Staffordshire terrier, Doberman pinscher) (49, 136). Currently, there is no empirical or epidemiological evidence to justify the required use of behavioral (re)tests, minimum canine age, or the exclusion of stipulated breeds in CAIs (40, 49).

Another welfare concern is the duration of CAI visits as sessions can vary from 15–120 min (31, 49). When more clients choose to attend sessions, the frequency of the interventions and the number of clients per session may increase, which can contribute to an increased workload, elevated stress levels in the dogs and possible undesirable behavior towards clients (31). In visiting programs, the transportation of animals might be a cause of elevated cortisol levels (137) and in residential CAIs where dogs and clients live together, clients may initiate an interaction at will, resulting in dogs being on duty 24 h/day with little time to rest (82). It is suggested that introducing a particular working-cue such as a bandana could help the dog to discriminate when the session starts.

## Conclusion

As universal rules do not exist to provide for the welfare of dogs in CAIs, which might cause health issues for CAI participants, we reviewed literature to understand existing knowledge and propose a solution. Based upon the different aspects discussed in the previous section, we propose the Welfare Quality Model as a risk awareness tool to ensure safety in CAIs. The model provides insights into how dog management, health, behavior, and the environment in which dogs work during CAIs may influence participants' welfare and health. It is proposed that the Welfare Quality Model may serve in a broader professional context as a shared communication tool for veterinarians, practitioners, and physicians in streamlining multidisciplinary co-operation concerning CAI risk assessment and prevention procedures. The benefits of CAIs must outweigh the risks, therefore an enhanced understanding of the interaction between welfare and health is crucial.

## Author Contributions

LM and CD-G analyzed the literature. LM and EW did the draft preparation. This commentary emerged from conversations between all authors over an extended period of time. All authors were involved equally in conceptualizing the topic, approve the final version of the manuscript, ensure the accuracy and integrity of the work, and agree to be accountable for all aspects of the work.

## Funding

Publishing cost was paid by SN's MIUR DOR.

## Conflict of Interest

The authors declare that the research was conducted in the absence of any commercial or financial relationships that could be construed as a potential conflict of interest.

## Publisher's Note

All claims expressed in this article are solely those of the authors and do not necessarily represent those of their affiliated organizations, or those of the publisher, the editors and the reviewers. Any product that may be evaluated in this article, or claim that may be made by its manufacturer, is not guaranteed or endorsed by the publisher.

## References

[1] Hill J Ziviani J Driscoll C Cawdell-Smith J. Can canine-assisted interventions affect the social behaviours of children on the autism spectrum? A Systematic Review Rev J Autism Dev Disord. (2019) 6:13–25. 10.1007/s40489-018-0151-7

[2] Stefanini MC Martino A Bacci B Tani F. The effect of animal-assisted therapy on emotional and behavioral symptoms in children and adolescents hospitalized for acute mental disorders. Eu J Integr Med. (2016) S187638201630018X–. 10.1016/j.eujim.2016.03.001

[3] Sánchez-Valdeón L Fernández-Martínez E Loma-Ramos S López-Alonso AI Bayón Darkistade E Ladera V. Canine-assisted therapy and quality of life in people with Alzheimer-type dementia: pilot study. Front Psychol. (2019) 10:1332. 10.3389/fpsyg.2019.01332 31244731 PMC6563674

[4] Ambrosi C Zaiontz C Peragine G Sarchi S Bona F. Randomized controlled study on the effectiveness of animal-assisted therapy on depression, anxiety, and illness perception in institutionalized elderly. Psychogeriatr. (2019) 19:55–64. 10.1111/psyg.12367 30221438

[5] Beck AM Katcher AH. Future directions in human-animal bond research. Am Behav Sci. (2003) 47:79–93. 10.1177/0002764203255214

[6] Odendaal JS Meintjes RA. Neurophysiological correlates of an affiliative behaviour between humans and dogs. Vet J. (2003) 165:296–301. 10.1016/S1090-0233(02)00237-X 12672376

[7] Handlin L Hydbring-Sandberg E Nilsson A Ejdebäck M Jansson A Uvnäs-Moberg K. Short-term interaction between dogs and their Owners: effects on oxytocin, cortisol, insulin and heart rate: an exploratory study. Anthrozoös. (2011) 24:301–15. 10.2752/175303711X13045914865385

[8] Miller SC Kennedy C DeVoe D Hickey M Nelson T Kogan L. An examination of changes in oxytocin levels in men and women before and after interaction with a bonded dog. Anthrozoös. (2009) 22:31–42. 10.2752/175303708X390455

[9] Powell L Guastella AJ McGreevy P Bauman A Edwards KM Stamatakis E. The physiological function of oxytocin in humans and its acute response to human-dog interactions: a review of the literature. J Vet Behav. (2019) 30:25–32. 10.1016/j.jveb.2018.10.008

[10] Glenk LM Kothgassner OD Stetina BU Palme R Kepplinger B Baran H. Therapy dogs' salivary cortisol levels vary during animal-assisted interventions. Anim Welf. (2013) 22:369–78. 10.7120/09627286.22.3.369

[11] Glenk LM Kothgassner OD Stetina BU Palme R Kepplinger B Baran H. Salivary cortisol and behavior in therapy dogs during animal-assisted interventions: a pilot study. J Vet Behav. (2014) 9:98–106. 10.1016/j.jveb.2014.02.005

[12] Ng ZY Pierce BJ Otto CM Buechner-Maxwell VA Siracusa C Werre SR. The effect of dog–human interaction on cortisol and behavior in registered animal-assisted activity dogs. Appl Anim Behav Sci. (2014) 159:69–81. 10.1016/j.applanim.2014.07.009

[13] Clark SD Martin F McGowan RTS Smidt JM Anderson R Wang L. Physiological state of therapy dogs during animal-assisted activities in an outpatient setting. Anim. (2020) 10:819. 10.3390/ani10050819 32397366 PMC7277909

[14] Clark SD Smidt JM Bauer BA. Welfare consideration: Salivary cortisol concentrations on frequency of therapy dog visits in an outpatient hospital setting: A pilot study. J Vet Behav. (2019) 30:88–91. 10.1016/j.jveb.2018.12.002

[15] Melco AL Goldman L Fine AH Peralta JM. Investigation of physiological and behavioral responses in dogs participating in animal-assisted therapy with children diagnosed with attention-deficit hyperactivity disorder. J Appl Anim Welf Sci. (2018) 23:10–28. 10.1080/10888705.2018.1536979 30376724

[16] Haubenhofer D Möstl E Kirchengast S. Cortisol concentrations in saliva of humans and their dogs during intensive training courses in animal-assisted therapy. Wien Tierärztl Mschr. (2005) 92:66–73.

[17] Silas HJ Binfet JT Ford AT. Therapeutic for all? Observational assessments of therapy canine stress in an on-campus stress-reduction program. J Vet Behav. (2019) 32:6–13. 10.1016/j.jveb.2019.03.009

[18] McCullough A Jenkins MA Ruehrdanz A Gilmer MJ Olson J Pawar A. Physiological and behavioral effects of animal-assisted interventions on therapy dogs in pediatric oncology settings. Appl Anim Behav Sci. (2018) 200:86–95. 10.1016/j.applanim.2017.11.014

[19] Corsetti S Ferrara M Natoli E. Evaluating stress in dogs involved in animal-assisted interventions. Anim. (2019) 9:833. 10.3390/ani9100833 31635094 PMC6827148

[20] Wesley MC Minatrea NB Watson JC. Animal assisted therapy in the treatment of substance dependence. Anthrozoös. (2009) 22:137–48. 10.2752/175303709X434167

[21] Jasperson RA. Animal-assisted therapy with female inmates with mental illness: a case example from a pilot program. J Offender Rehabil. (2010) 49:417–33. 10.1080/10509674.2010.499056

[22] Palestrini C Calcaterra V Cannas S Talamonti Z Papotti F Buttram D. Stress level evaluation in a dog during animal-assisted therapy in pediatric surgery. J Vet Behav. (2017) 17:44–9. 10.1016/j.jveb.2016.09.003

[23] Miller SL Serpell JA Dalton KR Waite KB Morris DO Redding LE. The importance of evaluating positive welfare characteristics and temperament in working therapy dogs. Front Vet Sci. (2022) 9:329. 10.3389/fvets.2022.844252 35445102 PMC9014261

[24] Black AF Chur-hansen A Winefield HR. Australian psychologists' knowledge of and attitudes towards animal-assisted therapy. Clin Psychol. (2011) 15:69–77. 10.1111/j.1742-9552.2011.00026.x

[25] Boyle SF Corrigan VK Buechner-Maxwell V Pierce BJ. Evaluation of risk of zoonotic pathogen transmission in a university-based animal assisted intervention (AAI) program. Front Vet Sci. (2019) 6:167. 10.3389/fvets.2019.00167 31214606 PMC6558202

[26] Gandenberger J Flynn E Moratto E Wendt A Morris KN. Molecular biomarkers of adult human and dog stress during canine-assisted interventions: a systematic scoping review. Anim. (2022) 12:651. 10.3390/ani12050651 35268219 PMC8909518

[27] Gussgard AM Weese JS Hensten A Jokstad A. Dog-assisted therapy in the dental clinic: Part A—Hazards and assessment of potential risks to the health and safety of humans. Clin Exp Dent Res. (2019) 5:692–700. 10.1002/cre2.240 31890307 PMC6934338

[28] Johnson RA Odendaal JS Meadows RL. Animal-assisted interventions research: Issues and answers. West J Nurs Res. (2002) 24:422–40. 10.1177/01945902024004009 12035914

[29] Chur-Hansen A McArthur M Winefield H Hanieh E Hazel S. Animal-assisted interventions in children's hospitals: a critical review of the literature. Anthrozoös. (2014) 27:5–18. 10.2752/175303714X13837396326251

[30] Friesen L. Exploring animal-assisted programs with children in school and therapeutic contexts. Early Child Educ J. (2010) 37:261–7. 10.1007/s10643-009-0349-5

[31] Marinelli L Normando S Siliprandi C Salvadoretti M Mongillo P. Dog assisted interventions in a specialized centre and potential concerns for animal welfare. Vet Res Commun. (2009) 33:93–5. 10.1007/s11259-009-9256-x 19578960

[32] Haubenhofer DK Kirchengast S. Physiological arousal for companion dogs working with their owners in animal-assisted activities and animal-assisted therapy. J Appl Anim Welf Sci. (2006) 9:165–72. 10.1207/s15327604jaws0902_5 16956319

[33] Haubenhofer DK Kirchengast S. Dog handlers' and dogs' emotional and cortisol secretion responses associated with animal-assisted therapy sessions. Soc Anim. (2007) 15:127–50. 10.1163/156853007X187090

[34] King C Watters J Mungre S. Effect of a time-out session with working animal-assisted therapy dogs. J Vet Behav Clin Appl Res. (2011) 6:232–38. 10.1016/j.jveb.2011.01.007

[35] Jalongo MR Astorino T Bomboy N. Canine visitors: the influence of therapy dogs on young children's learning and wellbeing in classrooms and hospitals. Early Child Educ J. (2004) 32:9–16. 10.1023/B:ECEJ.0000039638.60714.5f

[36] Schmidt V Mokrá M Demolli P Brüggen MC Möhrenschlager M. Allergologic pitfalls in animal-assisted interventions. Allergo J Int. (2022) 31:1–3. 10.1007/s40629-022-00206-9

[37] Forget S Pennequin V Agli O Bailly N. Brakes and levers to implement an animal-assisted intervention in nursing homes: preliminary study. Compl Ther Med. (2021) 56:102591. 10.1016/j.ctim.2020.102591 33197666

[38] Hediger K Hund-Georgiadis M. Animal-assisted therapy in the view of staff members before and after implementation in a rehabilitation clinic. Hum Anim Interact Bull. (2017) 5:61–73.

[39] Grové C Henderson L Lee F Wardlaw P. Therapy Dogs in Educational Settings: Guidelines and Recommendations for Implementation. Front Vet Sci. (2021) 8:655104. 10.3389/fvets.2021.655104 34169105 PMC8217446

[40] Lefebvre SL Waltner-Toews D Peregrine AS Reid-Smith R Hodge L Arroyo LG. Prevalence of zoonotic agents in dogs visiting hospitalized people in Ontario: implications for infection control. J of Hosp Inf. (2006) 62:458–66. 10.1016/j.jhin.2005.09.025 16466831

[41] Dalton KR Waite KB Ruble K Carroll KC DeLone A Frankenfield P. Risks associated with animal-assisted intervention programs: a literature review. Complement Ther Clin Pract. (2020) 39:101145. 10.1016/j.ctcp.2020.101145 32379677 PMC7673300

[42] Italian Italian Ministry of Health Italian National Guidelines for Animal Assisted Interventions (AAI). Agreement Between the Italian Government, the Regions and the Autonomous Provinces of Trento and Bolzano. (2015). Available online at: http://www.salute.gov.it/imgs/C_17_opuscoliPoster_276_allegato.pdf (accessed February 2022).

[43] Simonato M De Santis M Contalbrigo L Benedetti D Finocchi Mahne E Santucci VO. Farina L. The Italian agreement between the government and the regional authorities: national guidelines for AAI and institutional context. Peop Anim Int J Res Pract. (2018) 1:1–11.

[44] *Informationen über Therapiebegleithunde*. (2015). Available online at: https://www.vetmeduni.ac.at/de/therapiebegleithunde/informationen-ueber-therapiebegleithunde (accessed February 28, 2022).

[45] *Tierschutzgesetz - § 11 TierSchG*. (2006). Available online at: https://tierschutzgesetz.net/paragraph-11 (accessed February 28, 2022).

[46] Huss RJ. Legal and policy issues for animal assisted interventions with special populations. Appl Develop Sci. (2017) 21:217–22. 10.1080/10888691.2016.1231063

[47] Brelsford VL Dimolareva M Gee NR Meints K. Best practice standards in animal-assisted interventions: how the LEAD risk assessment tool can help. Anim. (2020) 10:974. 10.3390/ani10060974 32503309 PMC7341237

[48] Hartwig E Kjellstrand B Tyler J. What's important in canine-assisted intervention teams? An investigation of canine-assisted intervention program online screening tools. J Vet Behav. (2018) S1558787817302563. 10.1016/j.jveb.2018.09.004

[49] Serpell JA Kruger KA Freeman LM Griffin JA Ng ZY. Current standards and practices within the therapy dog industry: results of a representative survey of United States therapy dog organizations. Front Vet Sci. (2020) 7:35. 10.3389/fvets.2020.00035 32118059 PMC7020743

[50] Barker SB Gee NR. Canine-assisted interventions in hospitals: best practices for maximizing human and canine safety. Front Vet Sci. (2021) 8:615730. 10.3389/fvets.2021.615730 33869316 PMC8044758

[51] Ng Z Albright J Fine AH Peralta J. Our ethical and moral responsibility: ensuring the welfare of therapy animals. In: Fine AH, Editor. Handbook on Animal-Assisted Therapy: Theoretical Foundations and Guidelines for Animal-Assisted Interventions. 4th edition. San Diego, Elsevier (2015). p. 357–77.

[52] Moher D Liberati A Tetzlaff J Altman DG Grp P. Preferred reporting items for systematic reviews and meta-analyses: the PRISMA statement. J Clin Epidem. (2009) 62:1006–12. 10.1016/j.jclinepi.2009.06.005 19631508

[53] Page MJ McKenzie JE Bossuyt PM Boutron I Hoffmann TC Mulrow CD. The PRISMA 2020 statement: an updated guideline for reporting systematic reviews. BMJ. (2021) 372:n71. 10.1136/bmj.n71 33782057 PMC8005924

[54] PubMed. Available online at: http://www.ncbi.nlm.nih.gov/pubmed (accessed May 17, 2022).

[55] Google Scholar. Available online at: https://scholar.google.be/ (accessed May 17, 2022).

[56] Web of Science. Available online at: https://access.clarivate.com/login/ (accessed May 17, 2022).

[57] Harzing AW. Publish or Perish. Available online at: https://harzing.com/resources/publish-or-perish (accessed May 17, 2022).

[58] LaJoie KR. An evaluation of the effectiveness of using animals in therapy. Doctoral 251 dissertation. Louisville, KY: Spalding University (2003). p. 110.

[59] Kruger KA Serpell JA. Animal-assisted interventions in mental health: definitions and theoretical foundations. In: Fine AH, editor. Handbook on Animal-Assisted Therapy: Theoretical Foundations and Guidelines for Practice. San Diego: Elsevier (2010). p. 21–38.

[60] Canali E Keeling L. Welfare Quality® project: from scientific research to on farm assessment of animal welfare. Ital J Anim Sci. (2009) 8:900–3. 10.4081/ijas.2009.s2.900

[61] Blokhuis H Veissier I Jones B Miele M. The welfare quality® vision. In: Improving Farm Animal Welfare. Wageningen: Wageningen Academic Publishers (2013). p. 71–89.

[62] Glenk LM Foltin S. Therapy dog welfare revisited: a review of the literature. Vet Sci. (2021) 8:226. 10.3390/vetsci8100226 34679056 PMC8538106

[63] Winkle M Johnson A Mills D. Dog welfare, well-being and behavior: considerations for selection, evaluation and suitability for animal-assisted therapy. Anim. (2020) 10:2188. 10.3390/ani10112188 33238376 PMC7700550

[64] Evans N Gray C. The practice and ethics of animal-assisted therapy with children and young people: is it enough that we don't eat our co-workers? Brit J Soc Work. (2012) 42:600–17. 10.1093/bjsw/bcr091

[65] Lefebvre SL Golab GC Christensen E Castrodale L Aureden K Bialachowski A. Guidelines for animal-assisted interventions in health care facilities. Am J Infect Control. (2008) 36:78–85. 10.1016/j.ajic.2007.09.005 18313508

[66] Murthy R Bearman G Brown S Bryant K Chinn R Hewlett A. Animals in healthcare facilities: recommendations to minimize potential risks. Infect Control Hosp Epidemiol. (2015) 36:495–516. 10.1017/ice.2015.15 25998315

[67] Stogdale L. One veterinarian's experience with owners who are feeding raw meat to their pets. Can Vet J. (2019) 60:655–58. 31156268 PMC6515799

[68] Schlesinger DP Joffe DJ. Raw food diets in companion animals: a critical review. Can Vet J. (2011) 52:50–4. 21461207 PMC3003575

[69] Butowski CF Moon CD Thomas DG Young W Bermingham EN. The effects of raw-meat diets on the gastrointestinal microbiota of the cat and dog: a review. NZ Vet J. (2022) 70:1–9. 10.1080/00480169.2021.1975586 34463606

[70] Freeman LM Michel KE. Evaluation of raw food diet for dogs. J Am Vet Med Assoc. (2001) 218:705–9. 10.2460/javma.2001.218.705 11280399

[71] Freeman LM Chandler ML Hamper BA Weeth LP. Current knowledge about the risks and benefits of raw meat–based diets for dogs and cats. J Am Vet Med. (2013) 243:1549–58. 10.2460/javma.243.11.1549 24261804

[72] LeJeune JT Hancock DD. Public health concerns associated with feeding raw meat diets to dogs. J Am Vet Med. (2001) 219:1222–5. 10.2460/javma.2001.219.1222 11697364

[73] van Bree FPJ Bokken GCAM Mineur R Franssen F Opsteegh M van der Giessen JWB. Zoonotic bacteria and parasites found in raw meat-based diets for cats and dogs. Vet Rec. (2018) 182:50. 10.1136/vr.104535 29326391

[74] Strohmeyer RA Morley PS Hyatt DR Dargatz DA Scorza AV Lappin MR. Evaluation of bacterial and protozoal contamination of commercially available raw meat diets for dogs. J Am Vet Med Assoc. (2006) 228:537–42. 10.2460/javma.228.4.537 16478425

[75] Lefebvre SL Reid-Smith R Boerlin P Weese JS. Evaluation of the risks of shedding salmonellae and other potential pathogens by therapy dogs fed raw diets in Ontario and Alberta. Zoonoses Public Health. (2008) 55:470–80. 10.1111/j.1863-2378.2008.01145.x 18811908

[76] Hellgren J Hästö LS Wikström C Fernström LL Hansson I. Occurrence of Salmonella, Campylobacter, Clostridium and Enterobacteriaceae in raw meat-based diets for dogs. Vet Rec. (2019) 184:442. 10.1136/vr.105199 30833301

[77] Viegas FM Ramos CP Xavier RGC Lopes EO Júnior CAO. Fecal shedding of Salmonella spp, Clostridium perfringens, and Clostridioides difficile in dogs fed raw meat-based diets in Brazil and their owners' motivation. PLoS ONE. (2020) 15:e0231275. 10.1371/journal.pone.0231275 32287295 PMC7156072

[78] Santaniello A Varriale L Dipineto L Borrelli L Pace A Fioretti A. Presence of Campylobacterjejuni and *C. coli* in dogs under training for animal-assisted therapies. Int J Environ Res Public Health. (2021) 18:3717. 10.3390/ijerph18073717 33918252 PMC8038157

[79] Finley R Ribble C Aramini J Vandermeer M Popa M Litman M. The risk of salmonellae shedding by dogs fed Salmonella-contaminated commercial raw food diets. Can Vet J. (2007) 48:69–75. 17310625 PMC1716752

[80] Allen AR. One bacillus to rule them all? – Investigating broad range host adaptation in Mycobacterium bovis. Infect Genet Evol. (2017) 53:68–76. 10.1016/j.meegid.2017.04.018 28434972

[81] Sales MPU Taylor GM Hughes S Yates M Hewinson G Young DB. Genetic diversity among mycobacterium bovis isolates: a preliminary study of strains from animal and human sources. J Clin Microbiol. (2001) 39:4558–62. 10.1128/JCM.39.12.4558-4562.2001 11724883 PMC88587

[82] Meers LL Contalbrigo L Stevens VA Ulitina OM Laufer SJ Samuels WE. The state of animal-assisted interventions: COVID-19 safety protocols and ethical considerations. J Appl Anim Eth Res. (2021) 1:1–23. 10.1163/25889567-BJA10019

[83] Sanguansermsri P Jenkinson HF Thanasak J Chairatvit K Roytrakul S Kittisenachai S. Comparative proteomic study of dog and human saliva. PLoS ONE. (2018) 13:e0208317. 10.1371/journal.pone.0208317 30513116 PMC6279226

[84] Chomel BB Sun B. Zoonoses in the bedroom. Emerg Infect Dis. (2011) 17:167. 10.3201/eid1702.101070 21291584 PMC3298380

[85] Glenk LM. Current Perspectives on Therapy Dog Welfare in Animal-Assisted Interventions. Anim. (2017) 7:7. 10.3390/ani7020007 28157145 PMC5332928

[86] Lefebvre SL Peregrine AS Golab GC Gumley NR Waltner-Toews D Weese JS. veterinary perspective on the recently published guidelines for animal-assisted interventions in health-care facilities. J Am Vet Med Ass. (2008) 233:394–402. 10.2460/javma.233.3.394 18673025

[87] Hubálek Z Halouzka J Juricova Z. Host-seeking activity of ixodid ticks in relation to weather variables. J Vect Ecol. (2003) 28:159–65. 14714663

[88] Schulz M Mahling M Pfister K. Abundance and seasonal activity of questing Ixodes ricinus ticks in their natural habitats in southern Germany in 2011. J Vect Ecol. (2014) 39:56–65. 10.1111/j.1948-7134.2014.12070.x 24820556

[89] Johnson JL Ginsberg HS Zhioua E Whitworth UG Markowski D Hyland KE. Passive tick surveillance, dog seropositivity, and incidence of human Lyme disease. Vector-Borne Zoonotic Dis. (2004) 4:137–42. 10.1089/1530366041210710 15228814

[90] Pfeffer M Dobler G. Tick-borne encephalitis virus in dogs-is this an issue? Parasites Vectors. (2011) 4:1–8. 10.1186/1756-3305-4-59 21489255 PMC3094398

[91] Müller CS Tilgen W Pföhler C. Caterpillar dermatitis revisited: lepidopterism after contact with oak processionary caterpillar. Case Rep. (2011) 2011:bcr0320113967. 10.1136/bcr.03.2011.3967 22696629 PMC3082058

[92] Kaszak I Planellas M Dworecka-Kaszak B. Pine processionary caterpillar, Thaumetopoea pityocampa Denis and Schiffermüller, 1775 contact as a health risk for dogs. Ann Parasitol. (2015) 61:159–63. 10.17420/ap6103.02 26568988

[93] Maronna A Stache H Sticherling M. Lepidopterism - oak processionary caterpillar dermatitis: appearance after indirect out-of-season contact. JDDG: J Dtsch Dermatol. (2008) 6:747–50. 10.1111/j.1610-0387.2008.06652.x 18266862

[94] Chomel BB Boulouis HJ Maruyama S Breitschwerdt EB. Bartonella spp. in pets and effect on human health. Emerg Infect Dis. (2006) 12:389–94. 10.3201/eid1203.050931 16704774 PMC3291446

[95] Wilkerson MJ Bagladi-Swanson M Wheeler DW Floyd-Hawkins K Craig C Lee KW. The immunopathogenesis of flea allergy dermatitis in dogs, an experimental study. Vet Immunol Immunopathol. (2004) 99:179–92. 10.1016/j.vetimm.2004.02.006 15135984

[96] Rinaldi L Biggeri A Carbone S Musella V Catelan D Veneziano V. Canine faecal contamination and parasitic risk in the city of Naples (southern Italy). BMC Vet Res. (2006) 2:29. 10.1186/1746-6148-2-29 16995934 PMC1590007

[97] Simonato G Danesi P Frangipane di Regalbono A Dotto G Tessarin C Pietrobelli M. Surveillance of zoonotic parasites in animals involved in animal-assisted interventions (AAIs). Int J Environ Res Public Health. (2020) 17:7914. 10.3390/ijerph17217914 33126661 PMC7663587

[98] Traversa D Frangipane Di Regalbono A Di Cesare A La Torre F Drake J Pietrobelli M. Environmental contamination by canine geohelminths. Parasit Vectors. (2014) 7:67. 10.1186/1756-3305-7-67 24524656 PMC3929561

[99] Beck AJ Barber T McKenzie H Thorlakson J Dell C Keeping-Burke L. Perceptions and experiences of health care professionals and staff with animal-assisted interventions in health care settings: a qualitative systematic review protocol. JBI Evidence Synthesis. (2022) 20:924–30. 10.11124/JBIES-21-00151 35019870

[100] Maurelli MP Santaniello A Fioretti A Cringoli G Rinaldi L Menna LF. The presence of Toxocara eggs on dog's fur as potential zoonotic risk in animal-assisted interventions: a systematic review. Anim. (2019) 9:827. 10.3390/ani9100827 31635019 PMC6826609

[101] Jõgi NO Svanes C Siiak SP Logan E Holloway JW Igland J. Zoonotic helminth exposure and risk of allergic diseases: a study of two generations in Norway. Clin Exp Allergy. (2018) 48:66–77. 10.1111/cea.13055 29117468

[102] Daré LO Bruand PE Gérard D Marin B Lameyre V Boumédiène F. Associations of mental disorders and neurotropic parasitic diseases: a meta-analysis in developing and emerging countries. BMC Public Health. (2019) 19:1–12. 10.1186/s12889-019-7933-4 31805904 PMC6896488

[103] Gerardi F Santaniello A Del Prete L Maurelli MP Menna LF Rinaldi L. Parasitic infections in dogs involved in animal-assisted interventions. Ital J Anim Sci. (2018) 17:269–72. 10.1080/1828051X.2017.134493731635019

[104] Mariti C Bein S. Evaluation of dog welfare before and after a professional grooming session. Dog Behav. (2015) 1:8–15. 10.4454/DOGB.V1I1.002

[105] Bert F Gualano MR Camussi E Pieve B Voglino G Siliquini R. Animal assisted intervention: a systematic review of benefits and risks. Europ J Integr Med. (2016) 8:695–706. 10.1016/j.eujim.2016.05.005 32362955 PMC7185850

[106] Vredegoor DW Willemse T Chapman MD Heederik DJJ Krop EJM. Can f 1 levels in hair and homes of different dog breeds: lack of evidence to describe any dog breed as hypoallergenic. J Allergy Clin Immunol. (2012) 130:904–9. 10.1016/j.jaci.2012.05.013 22728082

[107] Toshihiro S Matsui T Suzuki K Chida K. Effect of pet removal on pet allergic asthma. Chest. (2005) 127:1565–71. 10.1378/chest.127.5.1565 15888829

[108] Barchas D Melaragni M Abrahim H Barchas E. The best medicine. Crit Care Nurs Clin North Am. (2020) 32:167–90. 10.1016/j.cnc.2020.01.002 32402314

[109] Nahm N Lubin J Lubin J Bankwitz BK Castelaz M Chen X. Therapy dogs in the emergency department. West J Emerg Med. (2012) 13:363–5. 10.5811/westjem.2011.5.6574 22942937 PMC3421977

[110] Linder DE Siebens HC Mueller MK Gibbs DM Freeman LM. Animal-assisted interventions: a national survey of health and safety policies in hospitals, eldercare facilities, and therapy animal organizations. Am J Infect Control. (2017) 45:883–7. 10.1016/j.ajic.2017.04.287 28673680 PMC5542869

[111] Lefebvre SL Reid-Smith RJ Waltner-Toews D Weese JS. Incidence of acquisition of methicillin-resistant Staphylococcus aureus, Clostridium difficile, and other health-care-associated pathogens by dogs that participate in animal-assisted interventions. J Am Vet Med Assoc. (2009) 234:1404–17. 10.2460/javma.234.11.1404 19480620

[112] Brodie SJ Biley FC Shewring M. An exploration of the potential risks associated with using pet therapy in healthcare settings. J Clin Nurs. (2002) 11:444–56. 10.1046/j.1365-2702.2002.00628.x 12100640

[113] Coughlan K Olsen KE Boxrud D Bender JB. Methicillin-resistant Staphylococcus aureus in resident animals of a long-term care facility. Zoonoses Public Health. (2010) 57:220–6. 10.1111/j.1863-2378.2009.01302.x 20042067

[114] Enoch D Karas J Slater J Emery M Kearns A Farrington M. Carriage in a pet therapy dog. J Hosp Infect. (2005) 60:186–8. 10.1016/j.jhin.2004.11.011 15866022

[115] Santaniello A Garzillo S Amato A Sansone M Fioretti A Menna LF. Occurrence of Pasteurella multocida in dogs being trained for animal-assisted therapy. Int J Environ Res Public Health. (2020) 17:6385. 10.3390/ijerph17176385 32887269 PMC7503519

[116] Santaniello A Sansone M Fioretti A Menna LF. Systematic review and meta-analysis of the occurrence of ESKAPE bacteria group in dogs, and the related zoonotic risk in animal-assisted therapy, and in animal-assisted activity in the health context. Int J Environ Res Publ Health. (2020) 17:3278. 10.3390/ijerph17093278 32397230 PMC7246456

[117] Ghasemzadeh I Namazi SH. Review of bacterial and viral zoonotic infections transmitted by dogs. J Med Life. (2015) 8:1–5. 28316698 PMC5319273

[118] Abbott Y Acke E Khan S Muldoon EG Markey BK Pinilla M. Zoonotic transmission of Streptococcus equi subsp. Zooepidemicus from a dog to a handler. J Med Microbiol. (2010) 59:120–3. 10.1099/jmm.0.012930-0 19745031

[119] Hardin P Brown J Wright ME. Prevention of transmitted infections in a pet therapy program: an exemplar. Am J Infect Control. (2016) 44:846–50. 10.1016/j.ajic.2016.01.007 27372389

[120] Fine AH Beck AM Ng Z. The state of animal-assisted interventions: addressing the contemporary issues that will shape the future. Int J Environ Res Public Health. (2019) 16:3997. 10.3390/ijerph16203997 31635430 PMC6843928

[121] Cavalli C Carballo F Dzik MV Bentosela M. Gazing as a help requesting behavior: a comparison of dogs participating in animal-assisted interventions and pet dogs. Anim Cogn. (2020) 23:141–7. 10.1007/s10071-019-01324-8 31720884

[122] Clark SD Smidt JM Bauer BA. Therapy dogs' and handlers' behavior and salivary cortisol during initial visits in a complex medical institution: a pilot study. Front Vet Sci. (2020) 7:854. 10.3389/fvets.2020.564201 33282927 PMC7691227

[123] Lefebvre SL Waltner-Toews D Peregrine A Reid-Smith R Hodge L Weese JS. Characteristics of programs involving canine visitation of hospitalized people in Ontario. Infect Control Hosp Epidemiol. (2006) 27:754–8. 10.1086/505099 16807853

[124] Zamir T. The moral basis of animal-assisted therapy. Soc Anim. (2006) 14:179–99. 10.1163/156853006776778770 16862727

[125] Hatch A. The view from all fours: A look at an animal-assisted activity program from the animals' perspective. Anthrozoös. (2007) 20:37–50. 10.2752/089279307780216632

[126] German AJ Blackwell E Evans M Westgarth C. Overweight dogs are more likely to display undesirable behaviours: results of a large online survey of dog owners in the UK. J Nutr Sci. (2017) 6:e14. 10.1017/jns.2017.5 28630691 PMC5468744

[127] Messam LL Kass PH Chomel BB Hart LA. The human–canine environment: a risk factor for non-play bites? Vet J. (2008) 177:205–15. 10.1016/j.tvjl.2007.08.020 17937998

[128] Rezac K Slama P. Human behavior preceding dog bites to the face. Vet J. (2015) 206:284–8. 10.1016/j.tvjl.2015.10.021 26598785

[129] Abuabara A. A review of facial injuries due to dog bites. Med Oral Patol Oral Cir Bucal. (2006) 11:348–50. 16816820

[130] Adamantos S Boag A. Thirteen cases of tetanus in dogs. Vet Rec. (2007) 161:298–302. 10.1136/vr.161.9.298 17766808

[131] Burkitt JM Sturges BK Jandrey KE Kass PH. Risk factors associated with outcome in dogs with tetanus: 38 cases (1987–2005). J Am Vet Med Assoc. (2007) 230:76–83. 10.2460/javma.230.1.76 17199496

[132] Pascual FB McGinley EL Zanardi LR Cortese MM Murphy TV. Tetanus surveillance—United States, 1998–2000. MMWR Surveill Summ. (2003) 52:1–8. 12825541

[133] Mills D Zulch H. Appreciating the role of fear and anxiety in aggressive behavior by dogs. Vet Focus. (2010) 20:44–9.

[134] Townsend L Gee NR. Recognizing and mitigating canine stress during animal assisted interventions. Vet Sci. (2021) 8:254. 10.3390/vetsci8110254 34822627 PMC8623698

[135] Acebes F Pellitero JL Muñiz-Diez C Loy I. Development of desirable behaviors in dog-assisted interventions. Anim. (2022) 12:477. 10.3390/ani12040477 35203184 PMC8868114

[136] Schalamon J Ainoedhofer H Singer G Petnehazy T Mayr J Kiss K. Analysis of dog bites in children who are younger than 17 years. Pediatrics. (2006) 117:e374–9. 10.1542/peds.2005-1451 16510617

[137] d'Angelo D d'Ingeo S Ciani F Visone M Sacchettino L Avallone L. Cortisol levels of shelter dogs in animal assisted interventions in a prison: an exploratory study. Anim. (2021) 11:345. 10.3390/ani11020345 33572936 PMC7911336

